# Hepta­carbonyl­bis­(μ-propane-1,3-di­thiol­ato)triiron(I,II)(2 *Fe*—*Fe*)

**DOI:** 10.1107/S1600536814004619

**Published:** 2014-03-08

**Authors:** Mingqiang Hu, Chengbing Ma, Huimin Wen, Honghua Cui, Changneng Chen

**Affiliations:** a State Key Laboratory of Structural Chemistry, Fujian Institute of Research on the Structure of Matter, Fuzhou, Fujian 350002, People’s Republic of China

## Abstract

The trinuclear title compound, [Fe_3_(C_3_H_6_S_2_)_2_(CO)_7_], is a mixed-valent Fe^I^/Fe^II^ complex and crystallizes with two mol­ecules of similar configuration in the asymmetric unit. The three Fe atoms in each mol­ecule display a bent arrangement [Fe—Fe—Fe = 156.22 (4) and 157.06 (3)°]. Both outer Fe^I^ atoms are six-coordinated in a distorted ocahedral coordination geometry defined by the bridging Fe^II^ atom, three carbonyl C atoms and two bridging S atoms. The coordination number of the central Fe^II^ atom is seven and includes bonding to the two outer Fe^I^ atoms, four bridging S atoms and one carbonyl C atom. The resulting coordination polyhedron might be described as a highly distorted monocapped trigonal prism. In the crystal packing, the mol­ecules exhibit a chain-like arrangement parallel to [100] and [001], and the resulting layers are stacked along [010]. The cohesion of the structure is dominated by van der Waals inter­actions.

## Related literature   

For models of the active sites of Fe—Fe hydrogenases, see: Tard *et al.* (2005 ▶); Best *et al.* (2007 ▶). For the structures of similar trinuclear mixed-valence iron complexes, see: Winter *et al.* (1982 ▶); Ghosh *et al.* (2011 ▶).
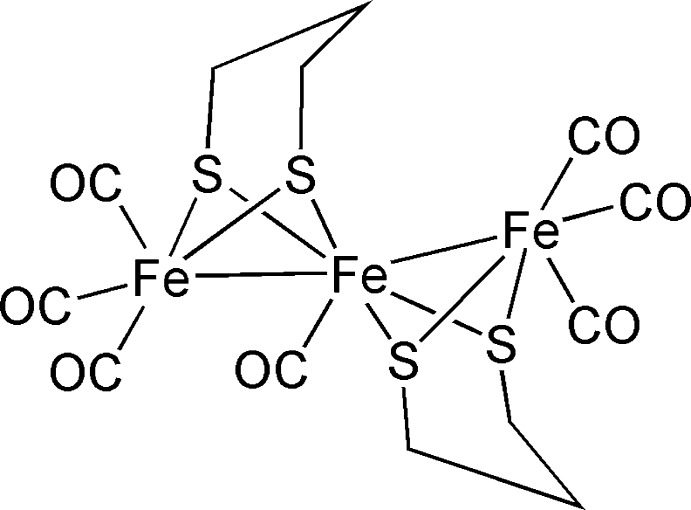


## Experimental   

### 

#### Crystal data  

[Fe_3_(C_3_H_6_S_2_)_2_(CO)_7_]
*M* *_r_* = 576.02
Orthorhombic, 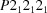

*a* = 10.251 (3) Å
*b* = 12.838 (4) Å
*c* = 30.915 (9) Å
*V* = 4068 (2) Å^3^
*Z* = 8
Mo *K*α radiation
μ = 2.55 mm^−1^
*T* = 293 K
0.25 × 0.20 × 0.12 mm

#### Data collection  

Rigaku Saturn724+ CCD diffractometer
Absorption correction: multi-scan (*CrystalClear*; Rigaku, 2007 ▶) *T*_min_ = 0.592, *T*_max_ = 1.000
35336 measured reflections
9231 independent reflections
8082 reflections with *I* > 2σ(*I*)
*R*_int_ = 0.071

#### Refinement  

*R*[*F* ^2^ > 2σ(*F* ^2^)] = 0.043
*wR*(*F* ^2^) = 0.056
*S* = 0.86
9231 reflections
488 parameters
H-atom parameters constrained
Δρ_max_ = 0.43 e Å^−3^
Δρ_min_ = −0.56 e Å^−3^
Absolute structure: Flack (1983 ▶), 4075 Friedel pairs
Absolute structure parameter: 0.016 (13)

### 

Data collection: *CrystalClear* (Rigaku, 2007 ▶); cell refinement: *CrystalClear*; data reduction: *CrystalClear*; program(s) used to solve structure: *SHELXS97* (Sheldrick, 2008 ▶); program(s) used to refine structure: *SHELXL97* (Sheldrick, 2008 ▶); molecular graphics: *SHELXTL* (Sheldrick, 2008 ▶) and *Mercury* (Macrae *et al.*, 2006 ▶); software used to prepare material for publication: *publCIF* (Westrip, 2010 ▶).

## Supplementary Material

Crystal structure: contains datablock(s) I. DOI: 10.1107/S1600536814004619/wm5007sup1.cif

Structure factors: contains datablock(s) I. DOI: 10.1107/S1600536814004619/wm5007Isup2.hkl

CCDC reference: 989211

Additional supporting information:  crystallographic information; 3D view; checkCIF report

## Figures and Tables

**Fig. 1.**
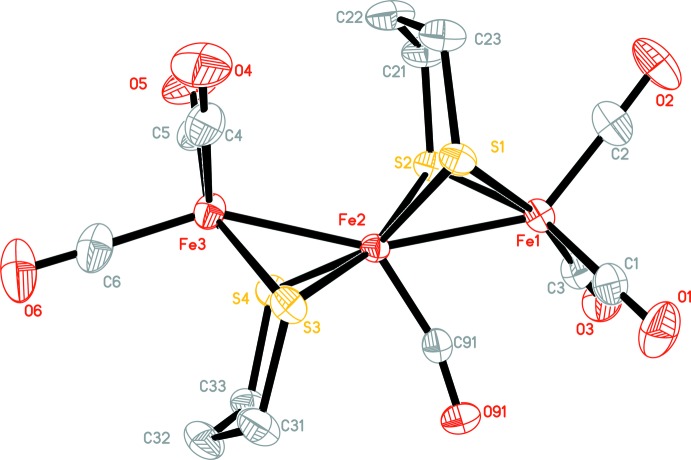
The molecular structure of (I) with displacement ellipsoids drawn at the 30% probability level. Only one of the two independent molecules is displayed, and H atoms have been omitted for clarity.

**Fig. 2.**
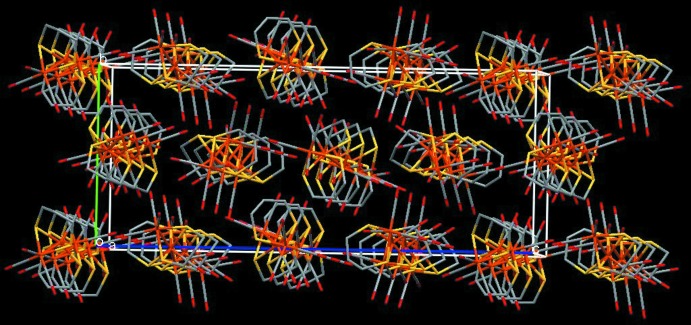
A packing diagram for (I), viewed along [100]. H atoms have been omitted for clarity.

## supplementary crystallographic information

### 1. Comment

The title compound, [Fe^I/II^_3_(C_3_H_6_S_2_)_2_(CO)_7_] (I), was prepared as a model compound for the active sites of Fe—Fe hydrogenases and structurally characterized by single-crystal X-ray diffraction. Such models have been reported for similar other compounds, *e.g.* Fe_4_[H_3_CC(CH_2_S)_3_]_2_(CO)_8_ (Tard *et al.*, 2005) and [Fe_4_(S(CH_2_)_2_S)_2_(CO)_2_(CO)_8_]_2_^2-^ (Best *et al.*, 2007).

Compound (I) crystallizes with two independent trinuclear iron molecules in the asymmetric unit. Formally, the three iron atoms in each molecule exhibit a mixed-valence, with the central Fe atom in oxidation state +II and the two lateral Fe atoms in oxidation state +I. Both molecules have a similar configuration and similar bond lengths and angles. The three Fe atoms display a slightly bent Fe_3_ core with Fe—Fe—Fe angles of 156.22 (4)° and 157.06 (3)°), respectively (Fig. 1). The outer Fe^I^ atoms are each six-coordinated by the central Fe^II^ atom (Fe—Fe: ≈ 2.56 Å), by three terminal carbonyl C atoms and by two bridging S atoms (Fe—S: ≈ 2.26 Å), leading to an overall distorted o­cta­hedral coordination environment. The central Fe^II^ atom is seven-coordinated. It is bound to the two lateral Fe atoms, to four bridging S atoms (Fe—S: ≈ 2.23-2.27 Å) and to one carbonyl group (Fe—C: ≈ 1.76 Å), completing a highly distorted monocapped trigonal-prismatic coordination environment.

In the crystal, the molecules are arranged in chains extending parallel to [100] and [001], resulting in a layer-like arrangement. These layers are stacked along [010] (Fig. 2). The main forces keeping the structure stabilized are van der Waals inter­actions.

Structures of similar trinuclear mixed-valence iron complexes have been reported by Winter *et al.* (1982) and Ghosh *et al.* (2011).

### 2. Experimental

Reactions were carried out under an atmosphere of purified nitro­gen, using standard Schlenk techniques. 5 g of Fe_3_(CO)_12_ were suspended in 200 mL of THF followed by the addition of two equivalents of 1,3-propane­dithiol. The reaction mixture was stirred at 343 k until its color changed from deep green to dark red. The reaction mixture was allowed to cool to room temperature and was filtered. The volume was reduced under vacuum to ca. 5 mL, and passed through a 25 X 3.0 cm column of silica gel, eluting with hexane. The eluting CH_2_Cl_2_ solution of a second run was collected and evaporated to dryness under vacuum. Red crystals of (I) were obtained from a hexane/CH_2_Cl_2_ solution at 253 K.

### 3. Refinement

H atoms bonded to C atoms were included in calculated positions with C—H = 0.97Å, and refined in a riding-model approximation with *U*_iso_(H) = 1.2*U*_eq_(C).

### Crystal data

**Table:**

[Fe_3_(C_3_H_6_S_2_)_2_(CO)_7_]	*F*(000) = 2304
*M**_r_* = 576.02	*D*_x_ = 1.881 Mg m^−^^3^
Orthorhombic, *P*2_1_2_1_2_1_	Mo *K*α radiation, λ = 0.710747 Å
Hall symbol: P 2ac 2ab	Cell parameters from 13590 reflections
*a* = 10.251 (3) Å	θ = 2.1–27.4°
*b* = 12.838 (4) Å	µ = 2.55 mm^−^^1^
*c* = 30.915 (9) Å	*T* = 293 K
*V* = 4068 (2) Å^3^	Prism, red
*Z* = 8	0.25 × 0.20 × 0.12 mm

### Data collection

**Table:**

Rigaku Saturn724+ CCD diffractometer	9231 independent reflections
Graphite Monochromator monochromator	8082 reflections with *I* > 2σ(*I*)
Detector resolution: 28.5714 pixels mm^-1^	*R*_int_ = 0.071
CCD_Profile_fitting scans	θ_max_ = 27.4°, θ_min_ = 2.5°
Absorption correction: multi-scan (*CrystalClear*; Rigaku, 2007)	*h* = −8→13
*T*_min_ = 0.592, *T*_max_ = 1.000	*k* = −16→16
35336 measured reflections	*l* = −40→40

### Refinement

**Table:**

Refinement on *F*^2^	Secondary atom site location: difference Fourier map
Least-squares matrix: full	Hydrogen site location: inferred from neighbouring sites
*R*[*F*^2^ > 2σ(*F*^2^)] = 0.043	H-atom parameters constrained
*wR*(*F*^2^) = 0.056	*w* = 1/[σ^2^(*F*_o_^2^) + (0.*P*)^2^] where *P* = (*F*_o_^2^ + 2*F*_c_^2^)/3
*S* = 0.86	(Δ/σ)_max_ = 0.001
9231 reflections	Δρ_max_ = 0.43 e Å^−^^3^
488 parameters	Δρ_min_ = −0.56 e Å^−^^3^
0 restraints	Absolute structure: Flack (1983), 4075 Friedel pairs
Primary atom site location: structure-invariant direct methods	Absolute structure parameter: 0.016 (13)

### Special details

**Table:**

Geometry. All e.s.d.'s (except the e.s.d. in the dihedral angle between two l.s. planes) are estimated using the full covariance matrix. The cell e.s.d.'s are taken into account individually in the estimation of e.s.d.'s in distances, angles and torsion angles; correlations between e.s.d.'s in cell parameters are only used when they are defined by crystal symmetry. An approximate (isotropic) treatment of cell e.s.d.'s is used for estimating e.s.d.'s involving l.s. planes.
Refinement. Refinement of *F*^2^ against ALL reflections. The weighted *R*-factor *wR* and goodness of fit *S* are based on *F*^2^, conventional *R*-factors *R* are based on *F*, with *F* set to zero for negative *F*^2^. The threshold expression of *F*^2^ > σ(*F*^2^) is used only for calculating *R*-factors(gt) *etc*. and is not relevant to the choice of reflections for refinement. *R*-factors based on *F*^2^ are statistically about twice as large as those based on *F*, and *R*- factors based on ALL data will be even larger.

### Fractional atomic coordinates and isotropic or equivalent isotropic displacement parameters (Å^2^)

**Table:**

	*x*	*y*	*z*	*U*_iso_*/*U*_eq_	
Fe1	1.04804 (6)	0.49655 (6)	0.03335 (2)	0.04003 (16)	
Fe2	0.81125 (6)	0.50119 (5)	0.059552 (18)	0.03290 (14)	
Fe3	0.61279 (7)	0.57503 (6)	0.09894 (2)	0.04215 (17)	
Fe4	0.36509 (6)	0.91038 (5)	0.19958 (2)	0.03942 (16)	
Fe5	0.12719 (6)	0.97257 (5)	0.198539 (19)	0.03369 (15)	
Fe6	−0.07197 (6)	1.07640 (5)	0.17247 (2)	0.03880 (16)	
S1	0.98615 (11)	0.53021 (9)	0.10260 (3)	0.0396 (3)	
S2	0.90097 (12)	0.62123 (9)	0.01479 (4)	0.0416 (3)	
S3	0.70965 (11)	0.42116 (10)	0.11404 (4)	0.0434 (3)	
S4	0.61473 (11)	0.51916 (9)	0.02945 (3)	0.0414 (3)	
S5	0.22266 (12)	0.87810 (10)	0.14501 (4)	0.0425 (3)	
S6	0.30258 (12)	1.07940 (10)	0.19239 (4)	0.0463 (3)	
S7	0.02210 (12)	1.09168 (9)	0.23814 (4)	0.0413 (3)	
S8	−0.06917 (11)	0.90355 (9)	0.18638 (3)	0.0376 (3)	
C1	1.1229 (5)	0.3756 (4)	0.05168 (16)	0.0531 (14)	
C2	1.1914 (5)	0.5763 (5)	0.03410 (15)	0.0612 (15)	
C3	1.0602 (5)	0.4602 (4)	−0.02280 (15)	0.0521 (13)	
C4	0.5998 (6)	0.7046 (5)	0.07693 (17)	0.0632 (17)	
C5	0.6824 (5)	0.6222 (4)	0.14889 (16)	0.0583 (15)	
C6	0.4492 (5)	0.5602 (5)	0.11754 (16)	0.0666 (17)	
C7	0.5117 (5)	0.9168 (4)	0.16823 (15)	0.0513 (13)	
C8	0.3729 (6)	0.7714 (4)	0.20949 (15)	0.0533 (14)	
C9	0.4340 (5)	0.9398 (4)	0.25218 (16)	0.0561 (14)	
C10	−0.0930 (5)	1.0443 (4)	0.11620 (15)	0.0564 (15)	
C11	0.0016 (5)	1.1998 (4)	0.16003 (16)	0.0530 (14)	
C12	−0.2357 (5)	1.1221 (4)	0.18135 (15)	0.0509 (14)	
C21	0.9519 (5)	0.7426 (3)	0.04054 (15)	0.0595 (15)	
H21A	1.0420	0.7560	0.0327	0.071*	
H21B	0.8997	0.7989	0.0288	0.071*	
C22	0.9411 (6)	0.7454 (4)	0.08884 (15)	0.0635 (17)	
H22A	0.8498	0.7369	0.0964	0.076*	
H22B	0.9674	0.8142	0.0985	0.076*	
C23	1.0183 (5)	0.6664 (4)	0.11395 (15)	0.0554 (14)	
H23A	1.0026	0.6783	0.1445	0.066*	
H23B	1.1102	0.6794	0.1087	0.066*	
C31	0.6108 (5)	0.3124 (4)	0.09543 (17)	0.0590 (15)	
H31A	0.6675	0.2641	0.0803	0.071*	
H31B	0.5770	0.2765	0.1207	0.071*	
C32	0.4959 (5)	0.3380 (4)	0.06570 (16)	0.0618 (16)	
H32A	0.4511	0.2736	0.0587	0.074*	
H32B	0.4351	0.3817	0.0814	0.074*	
C33	0.5323 (5)	0.3929 (4)	0.02356 (15)	0.0549 (14)	
H33A	0.4533	0.4034	0.0068	0.066*	
H33B	0.5886	0.3471	0.0070	0.066*	
C51	0.2710 (5)	0.9530 (4)	0.09738 (14)	0.0581 (16)	
H51A	0.3612	0.9366	0.0907	0.070*	
H51B	0.2184	0.9303	0.0731	0.070*	
C52	0.2581 (5)	1.0704 (4)	0.10154 (15)	0.0610 (15)	
H52A	0.2823	1.1016	0.0741	0.073*	
H52B	0.1670	1.0869	0.1066	0.073*	
C53	0.3386 (5)	1.1211 (4)	0.13688 (16)	0.0601 (15)	
H53A	0.3261	1.1959	0.1352	0.072*	
H53B	0.4299	1.1072	0.1311	0.072*	
C71	−0.0791 (4)	1.0318 (4)	0.28009 (12)	0.0501 (13)	
H71A	−0.0229	0.9900	0.2984	0.060*	
H71B	−0.1148	1.0871	0.2979	0.060*	
C72	−0.1902 (4)	0.9643 (4)	0.26522 (13)	0.0462 (12)	
H72A	−0.2515	1.0075	0.2494	0.055*	
H72B	−0.2351	0.9378	0.2906	0.055*	
C73	−0.1534 (4)	0.8733 (3)	0.23698 (14)	0.0472 (12)	
H73A	−0.2324	0.8352	0.2300	0.057*	
H73B	−0.0982	0.8271	0.2537	0.057*	
C91	0.8475 (4)	0.3835 (4)	0.03267 (15)	0.0481 (13)	
C92	0.1597 (4)	0.8936 (4)	0.24367 (14)	0.0435 (12)	
O1	1.1711 (4)	0.3008 (3)	0.06250 (13)	0.0823 (14)	
O2	1.2807 (4)	0.6289 (4)	0.03471 (13)	0.0925 (16)	
O3	1.0733 (4)	0.4380 (3)	−0.05810 (10)	0.0705 (11)	
O4	0.5945 (5)	0.7865 (3)	0.06323 (13)	0.0987 (17)	
O5	0.7256 (4)	0.6517 (3)	0.18024 (11)	0.0864 (15)	
O6	0.3438 (4)	0.5517 (4)	0.13040 (12)	0.0974 (17)	
O7	0.6055 (3)	0.9183 (3)	0.14762 (11)	0.0738 (12)	
O8	0.3816 (5)	0.6846 (3)	0.21508 (13)	0.0889 (14)	
O9	0.4785 (4)	0.9553 (3)	0.28507 (11)	0.0871 (15)	
O10	−0.1054 (4)	1.0259 (3)	0.08036 (10)	0.0889 (14)	
O11	0.0512 (4)	1.2781 (3)	0.15233 (12)	0.0840 (14)	
O12	−0.3387 (4)	1.1547 (3)	0.18505 (12)	0.0740 (13)	
O91	0.8444 (4)	0.3005 (3)	0.01739 (12)	0.0741 (12)	
O92	0.1563 (3)	0.8449 (3)	0.27565 (11)	0.0635 (11)	

### Atomic displacement parameters (Å^2^)

**Table:**

	*U*^11^	*U*^22^	*U*^33^	*U*^12^	*U*^13^	*U*^23^
Fe1	0.0352 (4)	0.0464 (4)	0.0385 (3)	−0.0028 (3)	−0.0006 (3)	0.0037 (3)
Fe2	0.0332 (3)	0.0332 (4)	0.0323 (3)	−0.0016 (3)	−0.0031 (3)	−0.0013 (3)
Fe3	0.0407 (4)	0.0513 (4)	0.0345 (3)	0.0075 (4)	−0.0042 (3)	−0.0054 (3)
Fe4	0.0354 (4)	0.0387 (4)	0.0441 (4)	−0.0018 (3)	−0.0002 (3)	−0.0029 (3)
Fe5	0.0325 (3)	0.0334 (4)	0.0351 (3)	−0.0030 (3)	0.0006 (3)	−0.0001 (3)
Fe6	0.0416 (4)	0.0383 (4)	0.0364 (3)	0.0038 (3)	−0.0007 (3)	−0.0005 (3)
S1	0.0433 (7)	0.0418 (7)	0.0338 (6)	−0.0068 (5)	−0.0075 (5)	0.0024 (5)
S2	0.0483 (8)	0.0385 (7)	0.0380 (6)	−0.0040 (6)	−0.0090 (5)	0.0068 (5)
S3	0.0407 (7)	0.0484 (8)	0.0410 (6)	−0.0056 (6)	−0.0036 (5)	0.0082 (6)
S4	0.0405 (7)	0.0478 (8)	0.0359 (6)	0.0011 (6)	−0.0081 (5)	−0.0030 (5)
S5	0.0394 (7)	0.0469 (8)	0.0411 (6)	−0.0073 (6)	0.0033 (5)	−0.0071 (5)
S6	0.0440 (7)	0.0348 (7)	0.0602 (8)	−0.0082 (6)	0.0065 (6)	−0.0024 (6)
S7	0.0463 (7)	0.0380 (7)	0.0397 (6)	−0.0006 (6)	−0.0011 (5)	−0.0070 (5)
S8	0.0366 (6)	0.0348 (6)	0.0414 (6)	−0.0041 (5)	0.0013 (5)	−0.0050 (5)
C1	0.045 (3)	0.063 (4)	0.051 (3)	0.000 (3)	0.007 (2)	0.002 (3)
C2	0.055 (3)	0.085 (4)	0.044 (3)	−0.012 (3)	−0.003 (3)	0.017 (3)
C3	0.045 (3)	0.059 (4)	0.053 (3)	0.001 (3)	−0.001 (2)	0.006 (3)
C4	0.071 (4)	0.066 (4)	0.053 (3)	0.021 (3)	−0.017 (3)	−0.015 (3)
C5	0.064 (4)	0.065 (4)	0.045 (3)	0.006 (3)	0.005 (3)	−0.002 (3)
C6	0.057 (4)	0.097 (5)	0.046 (3)	0.019 (4)	−0.006 (3)	−0.013 (3)
C7	0.045 (3)	0.056 (3)	0.054 (3)	−0.004 (3)	−0.006 (2)	−0.005 (3)
C8	0.063 (4)	0.045 (3)	0.052 (3)	0.001 (3)	0.007 (3)	0.000 (2)
C9	0.045 (3)	0.070 (4)	0.053 (3)	−0.002 (3)	0.006 (2)	−0.008 (3)
C10	0.060 (4)	0.059 (4)	0.051 (3)	0.010 (3)	0.001 (3)	0.008 (3)
C11	0.059 (4)	0.048 (3)	0.052 (3)	0.006 (3)	0.006 (3)	−0.002 (3)
C12	0.055 (4)	0.047 (3)	0.050 (3)	0.004 (3)	−0.004 (3)	0.007 (2)
C21	0.083 (4)	0.039 (3)	0.057 (3)	−0.003 (3)	−0.018 (3)	0.005 (2)
C22	0.088 (5)	0.035 (3)	0.067 (4)	−0.002 (3)	−0.026 (3)	−0.005 (3)
C23	0.072 (4)	0.044 (3)	0.050 (3)	−0.012 (3)	−0.014 (3)	−0.001 (2)
C31	0.050 (3)	0.053 (4)	0.075 (4)	−0.013 (3)	−0.007 (3)	0.014 (3)
C32	0.047 (4)	0.072 (4)	0.066 (4)	−0.021 (3)	−0.008 (3)	−0.005 (3)
C33	0.047 (3)	0.063 (4)	0.055 (3)	−0.014 (3)	−0.010 (2)	−0.017 (3)
C51	0.054 (3)	0.084 (5)	0.037 (3)	−0.007 (3)	0.008 (2)	−0.001 (3)
C52	0.057 (4)	0.074 (4)	0.052 (3)	0.007 (3)	0.018 (3)	0.018 (3)
C53	0.054 (4)	0.046 (3)	0.080 (4)	−0.007 (3)	0.015 (3)	0.018 (3)
C71	0.057 (3)	0.057 (3)	0.036 (2)	0.003 (3)	0.008 (2)	−0.007 (2)
C72	0.043 (3)	0.051 (3)	0.044 (3)	0.004 (2)	0.012 (2)	−0.002 (2)
C73	0.043 (3)	0.048 (3)	0.051 (3)	−0.004 (2)	0.007 (2)	0.003 (2)
C91	0.038 (3)	0.052 (3)	0.055 (3)	−0.001 (2)	0.006 (2)	−0.006 (3)
C92	0.035 (3)	0.048 (3)	0.048 (3)	0.001 (2)	−0.001 (2)	0.000 (2)
O1	0.078 (3)	0.078 (3)	0.091 (3)	0.036 (3)	0.006 (2)	0.020 (2)
O2	0.061 (3)	0.122 (4)	0.094 (3)	−0.044 (3)	−0.005 (2)	0.033 (3)
O3	0.080 (3)	0.085 (3)	0.047 (2)	0.005 (2)	0.004 (2)	−0.011 (2)
O4	0.159 (5)	0.052 (3)	0.085 (3)	0.039 (3)	−0.041 (3)	−0.003 (2)
O5	0.112 (4)	0.099 (4)	0.049 (2)	−0.017 (3)	−0.024 (2)	−0.019 (2)
O6	0.053 (3)	0.168 (5)	0.071 (3)	0.011 (3)	0.009 (2)	−0.010 (3)
O7	0.046 (2)	0.100 (3)	0.076 (3)	−0.009 (2)	0.015 (2)	−0.011 (2)
O8	0.125 (4)	0.046 (3)	0.095 (3)	0.011 (3)	0.013 (3)	0.009 (2)
O9	0.068 (3)	0.138 (4)	0.055 (2)	−0.015 (3)	−0.010 (2)	−0.023 (3)
O10	0.118 (4)	0.109 (4)	0.040 (2)	0.014 (3)	−0.010 (2)	−0.003 (2)
O11	0.114 (4)	0.049 (3)	0.088 (3)	−0.007 (3)	0.031 (3)	0.011 (2)
O12	0.054 (3)	0.087 (3)	0.081 (3)	0.020 (2)	0.000 (2)	0.004 (2)
O91	0.067 (3)	0.053 (3)	0.103 (3)	−0.007 (2)	0.009 (2)	−0.037 (2)
O92	0.052 (2)	0.079 (3)	0.060 (2)	0.005 (2)	0.0071 (19)	0.030 (2)

### Geometric parameters (Å, º)

**Table:**

Fe1—C2	1.791 (5)	C3—O3	1.136 (5)
Fe1—C3	1.802 (5)	C4—O4	1.135 (6)
Fe1—C1	1.823 (5)	C5—O5	1.131 (5)
Fe1—S2	2.2724 (14)	C6—O6	1.156 (6)
Fe1—S1	2.2743 (14)	C7—O7	1.154 (5)
Fe1—Fe2	2.5596 (11)	C8—O8	1.131 (5)
Fe2—C91	1.764 (5)	C9—O9	1.132 (5)
Fe2—S4	2.2310 (13)	C10—O10	1.140 (5)
Fe2—S3	2.2311 (13)	C11—O11	1.152 (6)
Fe2—S1	2.2637 (13)	C12—O12	1.142 (6)
Fe2—S2	2.2662 (13)	C21—C22	1.498 (6)
Fe2—Fe3	2.5534 (10)	C21—H21A	0.9700
Fe3—C6	1.783 (6)	C21—H21B	0.9700
Fe3—C4	1.802 (6)	C22—C23	1.503 (6)
Fe3—C5	1.806 (5)	C22—H22A	0.9700
Fe3—S3	2.2596 (15)	C22—H22B	0.9700
Fe3—S4	2.2648 (13)	C23—H23A	0.9700
Fe4—C7	1.790 (5)	C23—H23B	0.9700
Fe4—C8	1.812 (5)	C31—C32	1.529 (6)
Fe4—C9	1.813 (5)	C31—H31A	0.9700
Fe4—S5	2.2692 (14)	C31—H31B	0.9700
Fe4—S6	2.2734 (15)	C32—C33	1.528 (6)
Fe4—Fe5	2.5662 (11)	C32—H32A	0.9700
Fe5—C92	1.757 (4)	C32—H32B	0.9700
Fe5—S8	2.2310 (13)	C33—H33A	0.9700
Fe5—S7	2.2355 (13)	C33—H33B	0.9700
Fe5—S6	2.2693 (14)	C51—C52	1.519 (6)
Fe5—S5	2.2731 (14)	C51—H51A	0.9700
Fe5—Fe6	2.5679 (10)	C51—H51B	0.9700
Fe6—C11	1.796 (5)	C52—C53	1.516 (6)
Fe6—C12	1.799 (5)	C52—H52A	0.9700
Fe6—C10	1.801 (5)	C52—H52B	0.9700
Fe6—S7	2.2562 (14)	C53—H53A	0.9700
Fe6—S8	2.2604 (14)	C53—H53B	0.9700
S1—C23	1.814 (5)	C71—C72	1.503 (6)
S2—C21	1.826 (4)	C71—H71A	0.9700
S3—C31	1.818 (5)	C71—H71B	0.9700
S4—C33	1.836 (4)	C72—C73	1.506 (5)
S5—C51	1.827 (4)	C72—H72A	0.9700
S6—C53	1.835 (5)	C72—H72B	0.9700
S7—C71	1.830 (4)	C73—H73A	0.9700
S8—C73	1.829 (4)	C73—H73B	0.9700
C1—O1	1.131 (6)	C91—O91	1.165 (5)
C2—O2	1.138 (6)	C92—O92	1.170 (5)
			
C2—Fe1—C3	96.0 (2)	C21—S2—Fe1	107.53 (17)
C2—Fe1—C1	97.9 (2)	Fe2—S2—Fe1	68.66 (4)
C3—Fe1—C1	92.8 (2)	C31—S3—Fe2	112.00 (17)
C2—Fe1—S2	98.35 (19)	C31—S3—Fe3	111.15 (17)
C3—Fe1—S2	89.15 (16)	Fe2—S3—Fe3	69.30 (4)
C1—Fe1—S2	163.32 (17)	C33—S4—Fe2	111.44 (16)
C2—Fe1—S1	96.21 (17)	C33—S4—Fe3	111.68 (16)
C3—Fe1—S1	167.26 (16)	Fe2—S4—Fe3	69.21 (4)
C1—Fe1—S1	89.23 (16)	C51—S5—Fe4	109.19 (17)
S2—Fe1—S1	85.34 (5)	C51—S5—Fe5	115.02 (18)
C2—Fe1—Fe2	139.53 (19)	Fe4—S5—Fe5	68.80 (4)
C3—Fe1—Fe2	112.12 (16)	C53—S6—Fe5	114.44 (16)
C1—Fe1—Fe2	108.69 (16)	C53—S6—Fe4	108.24 (17)
S2—Fe1—Fe2	55.56 (4)	Fe5—S6—Fe4	68.79 (5)
S1—Fe1—Fe2	55.47 (3)	C71—S7—Fe5	111.96 (16)
C91—Fe2—S4	94.73 (16)	C71—S7—Fe6	111.03 (15)
C91—Fe2—S3	93.42 (16)	Fe5—S7—Fe6	69.74 (4)
S4—Fe2—S3	86.62 (5)	C73—S8—Fe5	111.49 (16)
C91—Fe2—S1	104.54 (16)	C73—S8—Fe6	111.43 (15)
S4—Fe2—S1	160.62 (5)	Fe5—S8—Fe6	69.74 (4)
S3—Fe2—S1	90.09 (5)	O1—C1—Fe1	178.7 (5)
C91—Fe2—S2	102.08 (16)	O2—C2—Fe1	178.4 (6)
S4—Fe2—S2	92.37 (5)	O3—C3—Fe1	177.1 (5)
S3—Fe2—S2	164.50 (6)	O4—C4—Fe3	178.4 (6)
S1—Fe2—S2	85.73 (5)	O5—C5—Fe3	179.7 (6)
C91—Fe2—Fe3	135.28 (15)	O6—C6—Fe3	178.5 (5)
S4—Fe2—Fe3	56.02 (4)	O7—C7—Fe4	178.2 (5)
S3—Fe2—Fe3	55.88 (4)	O8—C8—Fe4	177.8 (6)
S1—Fe2—Fe3	106.83 (4)	O9—C9—Fe4	178.0 (6)
S2—Fe2—Fe3	111.23 (5)	O10—C10—Fe6	178.6 (5)
C91—Fe2—Fe1	68.35 (15)	O11—C11—Fe6	178.5 (6)
S4—Fe2—Fe1	136.61 (4)	O12—C12—Fe6	176.2 (5)
S3—Fe2—Fe1	132.14 (4)	C22—C21—S2	115.7 (3)
S1—Fe2—Fe1	55.86 (4)	C22—C21—H21A	108.3
S2—Fe2—Fe1	55.79 (4)	S2—C21—H21A	108.4
Fe3—Fe2—Fe1	156.22 (4)	C22—C21—H21B	108.3
C6—Fe3—C4	98.7 (3)	S2—C21—H21B	108.3
C6—Fe3—C5	97.6 (2)	H21A—C21—H21B	107.4
C4—Fe3—C5	92.4 (2)	C21—C22—C23	117.4 (5)
C6—Fe3—S3	104.7 (2)	C21—C22—H22A	108.0
C4—Fe3—S3	156.6 (2)	C23—C22—H22A	108.0
C5—Fe3—S3	86.73 (17)	C21—C22—H22B	108.0
C6—Fe3—S4	106.28 (17)	C23—C22—H22B	108.0
C4—Fe3—S4	86.26 (16)	H22A—C22—H22B	107.2
C5—Fe3—S4	156.04 (18)	C22—C23—S1	117.0 (3)
S3—Fe3—S4	85.15 (5)	C22—C23—H23A	108.0
C6—Fe3—Fe2	149.7 (2)	S1—C23—H23A	108.0
C4—Fe3—Fe2	102.81 (19)	C22—C23—H23B	108.0
C5—Fe3—Fe2	102.55 (17)	S1—C23—H23B	108.0
S3—Fe3—Fe2	54.82 (4)	H23A—C23—H23B	107.3
S4—Fe3—Fe2	54.77 (4)	C32—C31—S3	117.1 (4)
C7—Fe4—C8	95.7 (2)	C32—C31—H31A	108.0
C7—Fe4—C9	98.6 (2)	S3—C31—H31A	108.0
C8—Fe4—C9	92.1 (2)	C32—C31—H31B	108.0
C7—Fe4—S5	98.40 (16)	S3—C31—H31B	108.0
C8—Fe4—S5	88.53 (17)	H31A—C31—H31B	107.3
C9—Fe4—S5	162.87 (16)	C33—C32—C31	115.0 (4)
C7—Fe4—S6	98.05 (18)	C33—C32—H32A	108.5
C8—Fe4—S6	165.65 (19)	C31—C32—H32A	108.5
C9—Fe4—S6	89.92 (18)	C33—C32—H32B	108.5
S5—Fe4—S6	85.43 (5)	C31—C32—H32B	108.5
C7—Fe4—Fe5	140.97 (16)	H32A—C32—H32B	107.5
C8—Fe4—Fe5	110.52 (19)	C32—C33—S4	115.8 (3)
C9—Fe4—Fe5	108.46 (16)	C32—C33—H33A	108.3
S5—Fe4—Fe5	55.67 (4)	S4—C33—H33A	108.3
S6—Fe4—Fe5	55.53 (4)	C32—C33—H33B	108.3
C92—Fe5—S8	94.36 (15)	S4—C33—H33B	108.3
C92—Fe5—S7	92.93 (16)	H33A—C33—H33B	107.4
S8—Fe5—S7	85.94 (5)	C52—C51—S5	115.5 (4)
C92—Fe5—S6	105.36 (16)	C52—C51—H51A	108.4
S8—Fe5—S6	160.16 (5)	S5—C51—H51A	108.4
S7—Fe5—S6	90.81 (5)	C52—C51—H51B	108.4
C92—Fe5—S5	100.88 (16)	S5—C51—H51B	108.4
S8—Fe5—S5	93.09 (5)	H51A—C51—H51B	107.5
S7—Fe5—S5	166.19 (5)	C53—C52—C51	116.1 (5)
S6—Fe5—S5	85.43 (5)	C53—C52—H52A	108.3
C92—Fe5—Fe4	68.29 (15)	C51—C52—H52A	108.3
S8—Fe5—Fe4	137.38 (5)	C53—C52—H52B	108.3
S7—Fe5—Fe4	131.59 (4)	C51—C52—H52B	108.3
S6—Fe5—Fe4	55.68 (4)	H52A—C52—H52B	107.4
S5—Fe5—Fe4	55.53 (4)	C52—C53—S6	116.1 (3)
C92—Fe5—Fe6	134.41 (15)	C52—C53—H53A	108.3
S8—Fe5—Fe6	55.67 (4)	S6—C53—H53A	108.3
S7—Fe5—Fe6	55.51 (4)	C52—C53—H53B	108.3
S6—Fe5—Fe6	106.85 (5)	S6—C53—H53B	108.3
S5—Fe5—Fe6	113.00 (4)	H53A—C53—H53B	107.4
Fe4—Fe5—Fe6	157.06 (3)	C72—C71—S7	117.1 (3)
C11—Fe6—C12	97.9 (2)	C72—C71—H71A	108.0
C11—Fe6—C10	92.6 (2)	S7—C71—H71A	108.0
C12—Fe6—C10	96.4 (2)	C72—C71—H71B	108.0
C11—Fe6—S7	86.36 (17)	S7—C71—H71B	108.0
C12—Fe6—S7	103.47 (16)	H71A—C71—H71B	107.3
C10—Fe6—S7	160.10 (17)	C71—C72—C73	115.8 (4)
C11—Fe6—S8	154.33 (17)	C71—C72—H72A	108.3
C12—Fe6—S8	107.63 (17)	C73—C72—H72A	108.3
C10—Fe6—S8	87.73 (17)	C71—C72—H72B	108.3
S7—Fe6—S8	84.77 (5)	C73—C72—H72B	108.3
C11—Fe6—Fe5	101.04 (17)	H72A—C72—H72B	107.4
C12—Fe6—Fe5	149.66 (15)	C72—C73—S8	116.7 (3)
C10—Fe6—Fe5	106.21 (16)	C72—C73—H73A	108.1
S7—Fe6—Fe5	54.75 (4)	S8—C73—H73A	108.1
S8—Fe6—Fe5	54.59 (4)	C72—C73—H73B	108.1
C23—S1—Fe2	114.61 (16)	S8—C73—H73B	108.1
C23—S1—Fe1	108.34 (17)	H73A—C73—H73B	107.3
Fe2—S1—Fe1	68.67 (4)	O91—C91—Fe2	165.4 (4)
C21—S2—Fe2	115.47 (16)	O92—C92—Fe5	166.8 (4)
